# Symposia

**DOI:** 10.1111/ene.70674

**Published:** 2026-06-26

## Saturday, June 27 2026

## EAN/MDS‐ES: Tremor syndromes: From pathophysiology to emerging treatment approaches

## SYMP01_1

### Pathophysiology of tremor syndromes

#### R. Helmich

##### Neurology Department, Radboud University Medical Centre, Donders Institute, Nijmegen, the Netherlands

Tremor syndromes are defined by clinical features, including tremor characteristics and accompanying symptoms. The pathophysiology of clinical tremor syndromes involves the mechanisms that give rise to the tremor. Knowledge about the pathophysiology of tremor is relevant, because it helps to understand clinical heterogeneity (e.g., in the response to treatment) and because it aids the development of new, mechanism‐guided therapies. Here, I will discuss the pathophysiology of the most common tremor syndromes, including Parkinson's tremor, essential tremor, and dystonic tremor. To this end, I will discuss evidence from studies that have used neuroimaging and neurophysiology outcomes, with or without interventions (e.g., pharmacological, stereotactic surgery). I will discuss how rhythmic activity in the cerebello‐thalamo‐cortical circuit plays a key role in almost all tremor syndromes. These activities are governed by differing cerebral mechanisms, both within the cerebello‐thalamo‐cortical circuit (e.g., cerebellar atrophy), and outside the cerebello‐thalamo‐cortical circuit (e.g., triggering circuits like the basal ganglia and counter‐acting circuits like recently discovered “anti‐tremor networks”).

**Disclosure:** Rick Helmich is consultant for Neurocrine Biosciences.

## SYMP01_2

### Diagnostic criteria and clinical approach to tremor syndromes

#### M. Stamelou

##### Hygeia Hospital, Athens, Greece

## SYMP01_3

### Pharmacological and other, non‐surgical, treatments of tremor syndromes

#### R. Erro

##### University of Salerno, Salerno, Italy

## SYMP01_4

### Surgical options for tremor syndromes: Clinical indications, efficacy and safety

#### Antonella Macerollo

##### The Walton Centre NHS Foundation Trust, Neurology, Liverpool, UK

## EAN/ESO: Future of medium size vessel occlusion stroke – Is there still a role for endovascular thrombectomy?

## SYMP02_1

### The role of blood‐based biomarkers in hyperacute risk stratification

#### M. Katan

##### University Hospital of Zurich, Neurology, Zurich, Switzerland

## SYMP02_2

### Use of imaging biomarkers for selection of patients who may still benefit from EVT?

#### R. Leker

##### Hadassah‐Hebrew University Medical Center, Neurology, Jerusalem, Israel

## SYMP02_3

### IA lytics use in patients with MeVO: The way forward

#### J. Kaesmacher

##### University of Bern, Neruoradiology

The treatment of Medium Vessel Occlusions (MeVO) represents on of the next frontiers in acute ischemic stroke intervention, moving beyond the established boundaries of large vessel mechanical thrombectomy (MT). While the 2015 “thrombectomy revolution” solidified MT as the gold standard for proximal occlusions, the management of more distal M2/M3, A2/A3, and P2/P3 segments remains a complex clinical challenge. This presentation evaluates the transition of intra‐arterial (IA) thrombolysis from a historical “rescue” therapy to a potential primary tool for achieving microvascular patency in primary or secondary MeVos. From initial IA Lytics trial in the pre‐EVT era the field is now reequipped with evidence for treatment of secondary MeVOs after LVO MT. Past and current evidence is reviewed and the role of patient selection, including intraoperative imaging is reviewed. The session concludes with a practical framework for clinical application, including dosing, timing and exact mode of administration, thereby providing a comprehensive roadmap for optimizing reperfusion in the distal cerebral vasculature.

**Disclosure:** TECNO Trial funded by Boehringer Ingelheim. Research contracts with Siemens and Cercare for intra‐operative Imaging.

## SYMP02_4

### New EVT devices and techniques for patients with MeVO

#### M. Psychogios

##### University Hospital Basel, Diagnostic and Interventional Neuroradiology, Basel, Switzerland

## EAN/ILAE Europe: Seizures, cognition and dementia: Hyper‐exciting times ahead

## SYMP03_1

### Size of the problem: Epidemiology of seizures in dementia

#### J. Zelano

##### Department of Neuroscience and Physiology, University of Gothenburg, Gothenburg, Sweden

## SYMP03_2

### Time flies faster: Seizures and the aging brain

#### K. Kobow

##### Department of Neuropathology, Universitätsklinikum Erlangen, Erlangen, Germany

## SYMP03_3

### Do antiseizure medications treat dementia: Lowering hyper‐excitability, improving cognition

#### M. Galovic

##### Department of Neurology, University Hospital Zurich, Zurich, Switzerland

## SYMP03_4

### Putting it all together: Best practice management of seizures in dementia

#### A. Sen

##### Department of Neurology, John Radcliffe Hospital, Oxford, UK

## EAN/ESRS: Can we reduce the sleep disorders burden with diagnostic and therapeutic innovations?

## SYMP04_1

### Epidemiology and economic burden of sleep disorders in Europe

#### C. Bassetti

##### Department of Neurology, University of Bern, Inselspital, Bern, Switzerland

## SYMP04_2

### Sleep revolution: Digitalisation of sleep diagnostics

#### E. Arnardottir

##### Department of Engineering, Department of Computer Science, Reykjavik University, Reykjavik, Iceland

## SYMP04_3

### New treatments for sleep disorders: From insomnia to hypersomnia

#### R. Fronczek

##### Department of Neurology, Leiden, Leiden University Medical Center, The Netherlands

## SYMP04_4

### When the world doesn’t sleep: How modern environments shape our heart and brain health

#### J. Reis

##### Hopital Universitaire de Hautepierre CHU Strasbourg, France

The studies of human biorhythms have become an important and emerging discipline in the sixties along with an interest for the sleep quality and duration. The media assumption of its decrease is debated. Sleep epidemiological studies are mostly based on self‐reported sleep duration via questionaries (seldom wearable devices). They were first conducted in WEIRD (Western, Educated, Industrialized, Rich and Democratic) countries before considering nowadays non‐WEIRD countries and even gather‐hunter populations. Finally, the first question concerns sleep quality: which are the optimal sleep duration and pattern? The perceived sleep efficacy as well as duration/pattern are both individual and cultural. We will question genetic, age and sex variability, the effects of societal, cultural as well the geographical latitude on the sleep duration's characteristics. The description of the Insufficient Sleep Syndrome, characterized by a quantitative deficit of sleep seems triggered by specific lifestyles. Initially observed among New‐York Yuppies, it seems nowadays related to some particular behaviors (e.g., work addiction). As well as imposed shift‐work, these conditions lead considering some recent societal constraints, our “modern environments”. These Zeitgebers include the effects of urbanization and technological revolution as well as lifestyle; they override the universal Zeitgeber which are light and the Day‐Night alternance. Understanding the health consequences of poor sleep quality, needs a global view of biorhythms as sleep may be considered as the perfect clinical marker of bio‐rhythmicity, or our Body Time. Amidst health risks, brain health, and cardio‐vascular and metabolic problems are prominent.

**Disclosure:** Nothing to disclose.

## EAN/WMS: Molecular therapies in neuromuscular disorders: From genes to clinical impact

## SYMP05_1

### Spinal muscular atrophies: From molecular mechanisms to therapeutic breakthroughs

#### S. Corti

##### Foundation IRCCS Ca' Granda Ospedale Maggiore Policlinico, University of Milan, Milan, Italy

Spinal muscular atrophy (SMA) is a severe neurodegenerative disorder caused by mutations in the SMN1 gene, resulting in progressive motor neuron loss and muscle atrophy. Over the past decade, SMA has transitioned from being an untreatable condition to a model of successful molecular therapy, fundamentally transforming patient outcomes and quality of life.

This lecture will provide an integrated overview of the molecular pathogenesis of SMA and the revolutionary therapeutic strategies that have emerged. Gene replacement therapy using onasemnogene abeparvovec, an AAV9‐mediated approach delivering functional SMN1 gene copies, has demonstrated remarkable efficacy, particularly when administered in presymptomatic or early symptomatic stages. The lecture will discuss clinical trial data, long‐term outcomes, and practical considerations for implementation.

Splicing modulators, including nusinersen and risdiplam, represent groundbreaking RNA‐targeted approaches that enhance SMN2 exon 7 inclusion to increase functional SMN protein production. Comparative analysis of antisense oligonucleotide and small‐molecule strategies will be presented, highlighting their distinct pharmacological profiles and clinical applications across different SMA phenotypes.

The critical importance of therapy timing and personalization will be emphasized, drawing from newborn screening programs and natural history studies that underscore the window of therapeutic opportunity. Patient selection criteria, biomarkers for treatment response monitoring, and individualized treatment algorithms based on SMN2 copy number and disease severity will be discussed.

Finally, new and emerging therapies, including next‐generation gene therapies, combination treatment strategies, and neuroprotective approaches targeting muscle and neuronal pathways beyond SMN restoration, will be explored as the future frontier of SMA management.

**Disclosure:** Participation in SAB of Novartis, Roche, Biogen.

## SYMP05_2

### Advances in molecular therapies for muscular dystrophies: Targeting the underlying defects

#### G. Comi

##### Foundation IRCCS Ca' Granda Ospedale Maggiore Policlinico, University of Milan, Milan, Italy

Muscular dystrophies represent a heterogeneous group of inherited disorders characterized by progressive muscle weakness and degeneration. Recent advances in molecular medicine have revolutionized the therapeutic landscape, shifting from purely symptomatic management toward disease‐modifying interventions that address the underlying genetic defects.

This lecture will provide a comprehensive overview of cutting‐edge molecular therapies currently in development or clinical use. Gene and RNA‐based therapies, including exon‐skipping antisense oligonucleotides, stop‐codon read‐through agents, and adeno‐associated virus (AAV)‐mediated gene replacement strategies, have demonstrated promising results in conditions such as Duchenne muscular dystrophy and limb‐girdle muscular dystrophies. The lecture will discuss the mechanistic principles, clinical trial outcomes, and real‐world evidence supporting these approaches.

Targeted molecular approaches will be explored, focusing on precision medicine strategies tailored to specific genetic mutations and disease subtypes. Particular attention will be given to the development of personalized therapeutic protocols and the importance of genetic diagnosis in treatment selection.

Finally, the lecture will address current challenges and future directions, including immunological responses to viral vectors, limited tissue biodistribution, long‐term safety concerns, and the need for biomarkers to monitor therapeutic efficacy. Emerging technologies, such as CRISPR‐based gene editing and next‐generation delivery systems, will be discussed as potential solutions to overcome existing limitations.

**Disclosure:** Participations to SAB of Roche, Novartis, Biogen, Sarepta.

## SYMP05_3

### Emerging therapies in ALS: The use of Innovative Human In Vitro Systems

#### J. Pasterkamp

##### University Medical Center Utrecht, Utrecht, The Netherlands

## SYMP05_4

### Human pluripotent stem cell‐derived models for the evaluation of therapeutics in neuromuscular diseases

#### C. Martinat

##### INSERM UMR861 I‐Stem, Corbeil‐Essonnes, France

## Monday, June 29 2026

## Innovations in Neurology: From Brain Machine Interface to AI

## SYMP06_1

### Adaptive deep brain stimulation for movement disorders

#### A. Kühn

##### Charité Universitätsmedizin Berlin, Neurology, Berlin, Germany

## SYMP06_2

### Advances in non‐invasive brain stimulation

#### F. Hummel

##### Swiss Federal Institute of Technology Lausanne (EPFL), Neuro‐X Institute, Geneva, Switzerland

## SYMP06_3

### Applying AI in clinical neurology

#### J. Teo

##### Kings College Hospital, London, UK

## SYMP06_4

### Interacting with robots

#### A. Rosenthal‐von der Pütten

##### RWTH Aachen University, Chair Individual and Technology, Aachen, Germany

## EAN/MDS‐ES: Dementia with Lewy bodies: Current and future perspectives

## SYMP07_1

### Diagnosis of DLB within the framework of neuronal Lewy body diseases

#### E. Lemstra

##### Amsterdam University Medical Center, Neurology, Amsterdam, The Netherlands

## SYMP07_2

### Role of imaging in the assessment of neurodegeneration, longitudinal monitoring and target engagement

#### D. Ferreira

##### Karolinska Institutet, Blickagången 16 (byggnad NEO), Huddinge, Sweden

## SYMP07_3

### Neurophysiological biomarkers for disease stratification and longitudinal assessment

#### L. Bonanni

##### Università degli Studi “G. d'Annunzio” Chieti – Pescara, Chieti, Italy

## SYMP07_4

### Treatment pathways, current and future perspectives

#### D. Arsland

##### King's College London, London, UK

## Which are the best medicines to treat migraine patients: New international preventive and acute therapies guidelines

## SYMP08_1

### Evidence based guidelines for preventive therapies

#### P. Pozo‐Rosich

##### Vall d'Hebron University Hospital, Unidad de Cefalea – Hospital de la Dona (2ª planta)Ps., Barcelona, Spain

## SYMP08_2

### Evidence based guidelines for acute therapies

#### V. Romanenko

##### International Association for the Study of Pain, Kyiv, Ukraine

## SYMP08_3

### What to do in clinical practice: Expert consensus on acute and preventive therapies for migraine

#### S. Sacco

##### University of L'Aquila, Italy

## SYMP08_4

### Central mechanism of CGRP target therapies: Will they modify migraine outcome?

#### M. de Tommaso

##### Policlinico bari, Neurology, Bari, Italy

## EAN/ECTRIMS: Integrating the new revision of the McDonald criteria into clinical practice

## SYMP09_1

### Overview of the 2024 revision of the McDonald criteria

#### M. Tintoré

##### Centre d'Esclerosi Mútiple de Catalunya (Cemcat), Barcelona, Spain

## SYMP09_2

### Assessment of optic nerve involvement

#### C. Granziera

##### University of Basel, Basel, Switzerland

## SYMP09_3

### MRI markers: Central vein sign and paramagnetic rim lesions

#### P. Preziosa

##### IRCCS San Raffaele Scientific Institute, Milan, Italy

## SYMP09_4

### Application and challenges in the clinical scenario of revised McDonald criteria

#### B. Stankoff

##### Hopital Saint Antoine, Soirbonne University, Paris, France

## EAN/EANO: The changing landscape of neuro‐oncology: New frontiers of treatments

## SYMP10_1

### Anatomo‐physiological basis of the blood–brain barrier in brain tumours

#### A. Di Stefano

##### Spedali Riuniti di Livorno, Livorno, Italy

The blood–brain barrier (BBB) represents one of the most distinctive anatomical and physiological features of the central nervous system and plays a pivotal role in the pathogenesis, diagnosis, and treatment of brain tumours. While the BBB is essential for maintaining neural homeostasis by regulating molecular trafficking between the systemic circulation and the brain parenchyma, it also constitutes the principal obstacle to the effective delivery of therapeutic agents to intracranial neoplasms. Indeed, many potentially active anticancer drugs fail to demonstrate clinical efficacy not because of inadequate target engagement, but because insufficient concentrations reach the tumour tissue. Understanding the anatomy and physiology of the BBB is therefore fundamental to modern neuro‐oncology. This lecture will review the structural organization of the BBB, focusing on the neurovascular unit, a dynamic multicellular system composed of endothelial cells, pericytes, astrocytic end‐feet, neurons, microglia, and extracellular matrix components. Particular attention will be given to the specialized properties of brain endothelial cells, including tight junction proteins such as claudin‐5, occludin, and zonula occludens‐1, the suppression of transcytosis pathways, and the expression of active efflux transporters including P‐glycoprotein and breast cancer resistance protein. Together, these mechanisms tightly regulate molecular exchange, preserve ionic and metabolic homeostasis, and limit immune cell trafficking within the central nervous system.

The presentation will then examine how these physiological barriers are altered in brain tumours. In gliomas, particularly glioblastoma, the normal BBB undergoes profound but heterogeneous remodeling, giving rise to the so‐called blood–tumour barrier (BTB). Contrary to the common perception of a uniformly disrupted barrier, permeability changes within gliomas are highly variable across different tumour regions. Tumour angiogenesis, driven in part by vascular endothelial growth factor (VEGF), results in abnormal endothelial differentiation, pericyte dysfunction, and increased vascular permeability. Nevertheless, infiltrative tumour cells frequently invade surrounding brain tissue where the BBB remains structurally and functionally intact. This preserved barrier at the tumour margins is a major contributor to treatment failure and disease recurrence.

The BBB in brain metastases exhibits distinct biological features, often showing greater permeability than primary gliomas, although substantial inter‐ and intra‐tumour heterogeneity persists. These alterations have important clinical consequences, contributing to vasogenic edema, increased intracranial pressure, and the radiological appearance of contrast enhancement on magnetic resonance imaging (MRI).

The lecture will also discuss current approaches for assessing BBB integrity in clinical practice. Gadolinium‐enhanced MRI remains the most widely used surrogate marker of BBB disruption; however, contrast enhancement does not necessarily reflect the full extent of tumour infiltration. Advanced imaging techniques, including dynamic contrast‐enhanced and dynamic susceptibility contrast MRI, provide additional insights into vascular permeability and tumour physiology, although their ability to predict drug penetration remains limited.

Finally, the therapeutic implications of BBB and BTB biology will be explored. The differential permeability of the BBB explains why certain agents, such as temozolomide, achieve clinically meaningful concentrations within the brain, whereas many targeted therapies do not. Emerging strategies aimed at overcoming or bypassing the BBB include convection‐enhanced delivery, focused ultrasound combined with microbubbles, nanoparticle‐based drug delivery systems, and local cellular immunotherapies such as CAR‐T approaches. Increasingly, the heterogeneity of the BTB is being recognized not only as an obstacle but also as a potential therapeutic opportunity.

A deeper understanding of BBB anatomy and physiology is essential for the development of next‐generation neuro‐oncological therapies. Future advances may enable patient‐specific characterization of BBB status, allowing treatment selection to be guided not only by tumour biology but also by the dynamic properties of the tumour microenvironment and its vascular barriers.

**Disclosure:** Nothing to disclose.

## SYMP10_2

### Disrupting BBB to enhance treatment delivery

#### A. Idbaih

##### Sorbonne Université, AP‐HP/La Pitié Salpêtrière, ICM

The blood–brain barrier (BBB) represents a major obstacle to drug delivery in treatment of central nervous system (CNS) disorders including brain cancer such as glioblastoma. The BBB is a highly selective biophysical and biochemical barrier that regulates molecular transport from the blood flow stream to the brain parenchyma.

In the setting of brain tumors, several strategies have been developed to overcome, bypass, and/or disrupt the BBB, to improve the bioavailability and the efficacy of therapeutic agents against tumor cells.

First, strategies aim at increasing drug delivery using systemic or local routes. High‐dose intravenous chemotherapy has significantly improved outcomes in selected brain malignancies. Intra‐arterial delivery approaches have also been explored. In addition, locally implanted biodegradable agents within the surgical cavity have demonstrated antitumor efficacy in recurrent glioblastoma.

Second, approaches focus on increasing drug penetration through drug or BBB modulation. Drug modulation strategies include several modalities, among which nanoparticle encapsulation is one of the most advanced, with encouraging signals of efficacy. Finally, direct BBB modulation represents a promising approach, including inhibition of efflux pumps and transient disruption of tight junctions. Ultrasound‐mediated BBB opening is currently being evaluated in a phase III clinical trial in brain tumors.

Overall, the BBB remains a major limiting factor in the treatment of CNS diseases, including brain tumors. Strategies aimed at overcoming, bypassing, or disrupting the BBB have the potential to enhance local therapeutic efficacy while reducing systemic toxicity.

**Disclosure:** Travel funding: Carthera, Leo Pharma, Novocure; research grants: Transgene, Sanofi, Servier, Amgen and Nutritheragene; consulting: Novocure, Novartis, Polytone Laser, Leo Pharma, and Boehringer Ingelheim.

## SYMP10_3

### Isocitrate dehydrogenase inhibitors for glioma: Efficacy and implications for service delivery

#### C. McBain

##### The Christie NHS FT, Department of Clinical Oncology, Manchester, UK

## SYMP10_4

### Quality of life and neurocognitive functions in low grade glioma patients

#### F. Boele

##### Patient‐Centred Outcomes Research Group, University of Leeds, Leeds, UK

